# Unveiling the material basis of Shenshuaifu granule and its therapeutic mechanism in chronic renal failure: a combined approach of high-resolution mass spectrometry and *in silico* technology

**DOI:** 10.3389/fchem.2025.1563598

**Published:** 2025-08-20

**Authors:** Qingbao Huang, Leyao Xian, Xiansheng Song, Dawei Zou, Junqi Chen, Yue Chen, Wanquan Li, Shangbin Zhang, Huichao Liang

**Affiliations:** ^1^ Shenzhen Key Laboratory of Hospital Chinese Medicine Preparation, Shenzhen Traditional Chinese Medicine Hospital, The Fourth Clinical Medical College of Guangzhou University of Chinese Medicine, Shenzhen, China; ^2^ Chinese Medicine Guangdong Laboratory, The Second Affiliated Hospital of Guangzhou University of Chinese Medicine, Guangzhou, China

**Keywords:** chronic renal failure, Shenshuaifu granule, AGE-RAGE signaling pathway, UHPLC-QE-MS, flavonoids

## Abstract

**Background:**

Chronic renal failure (CRF) is a serious state of continuous development in various chronic renal diseases. Shenshuaifu granule is a hospital preparation of Chinese medicines used at Shenzhen Traditional Chinese Medicine Hospital. It has been reported to delay the progression of renal failure in clinical application, but its material basis and mechanism are still unclear.

**Methods:**

In this study, ultra-high-performance liquid chromatography tandem with Q-Exactive-Orbitrap mass spectrometry (UHPLC-QE-MS), network pharmacology, and molecular docking technology were used to rapidly explore the plant metabolites of Shenshuaifu granule and its potential mechanisms.

**Results:**

The results showed that a total of 56 plant metabolites were identified from Shenshuaifu granule, including nine prototype metabolites detected in rat plasma after administration. Through network pharmacology and molecular docking technology, the plant metabolites that enter the blood from Shenshuaifu granule may regulate key targets, such as TNF, SRC, STAT3, EGFR, BCL2, JUN, and PTGS2. The GO and KEGG enrichment analysis showed that the AGE-RAGE signaling pathway, HIF-1 signaling pathway, and TNF signaling pathway were the main mechanisms of Shenshuaifu granule in the treatment of CRF.

**Conclusion:**

Flavonoids and quinones in Shenshuaifu granule are potential active plant metabolites, suggesting anti-inflammatory, anti-glomerulosclerosis, and fibrosis effects on CRF.

## 1 Introduction

Chronic renal failure (CRF) is the progressive renal parenchymal damage caused by various chronic kidney diseases, which is characterized by renal atrophy and loss of basic function, leading to the normal discharge of metabolic waste and excess water in the body, and causing a series of metabolic disorders and clinical syndrome with multiple system symptoms (Dai et al., 2021; Fang et al., 2023; Zou et al., 2020). According to the data provided by the International Society of Nephrology, the annual incidence of CRF in the population was approximately 98–198 cases per million people. With the improvement of people’s living standards, the incidence of CRF is increasing every year, and the cost of treatment is also increasing, which puts significant pressure on national medical budgets (Kotur-Stevuljević et al., 2013). The treatment strategies of CRF include drug therapy, hemodialysis, peritoneal dialysis, and kidney transplantation. However, dialysis and kidney transplantation are not only expensive but also have limited donor resources, which has brought significant economic pressure to many patients. Therefore, for patients with early- and middle-stage CRF, drug therapy has become the main treatment method to control the development of the disease (Tu et al., 2024).

Shenshuaifu granule is an in-hospital preparation of the Shenzhen Traditional Chinese Medicine Hospital, which is composed of *Astragalus membranaceus* root, *Salvia miltiorrhiza* root and rhizome, *Lycium chinense* fruit, *Leonurus japonicus* herb, *poria cocos* sclerotium, *Ilex asprella* root, *Alisma orientale* rhizome, and *Rheum palmatum* root and rhizome (Li et al., 2011). It has certain therapeutic effects in delaying the progression of CRF (Zhang and Cao, 2020). Animal experiments have revealed that Shenshuaifu granule attenuated acute kidney injury by inhibiting ferroptosis and inflammation by the p53/SLC7A11/GPX4 pathway (Jin et al., 2023a) and the TLR4/MyD88/NF-kappa B pathway (Jin et al., 2023b), respectively.

In addition, some reports on the herbs comprising Shenshuaifu granule can also provide a reference for the mechanism study of the granule. For example, *L. chinense* fruit and its extract showed therapeutic potential for kidney disease through improving renal function indicators, antioxidant anti-inflammatory properties, regulating oxidative stress and antioxidant enzymes, and providing renal tissue protection (Olatunji et al., 2018; Park et al., 2021). *S. miltiorrhiza* root and rhizome reduced ROS level and alleviated renal injury by regulating the NADPH oxidase signaling pathway and also regulated the signaling pathways related to renal fibrosis and inflammation, including the TGF-β/Smad signaling pathway, which has a significant protective effect on CRF (Cai et al., 2018). *Poria cocos* sclerotium improved kidney diseases through a variety of molecular mechanisms, including activating RAS, MMP, AHR, AMPK, TPH-1, aquaporin-2, and SGK1 molecules, and regulating the TGF-β1/Smad, Wnt/β-catenin, IκB/NF-κB, and Keap1/Nrf2 signaling pathways (Guo et al., 2024).

As is known, Chinese medicines have multi-component, multi-target, and multi-channel characteristics in the treatment of diseases. The research on Shenshuaifu granule mainly focuses on clinical efficacy observation and pharmacological research. However, it remains a mystery as to which specific chemical components in the granule exert their particular therapeutic effect. Insufficient research on the material basis restricts the scientificity of its rational application in clinical practice.

High-resolution mass spectrometry enables rapid, accurate, and high-throughput qualitative analysis of complex chemical compounds, making it a powerful tool for identifying plant-derived metabolites in Shenshuaifu granule. However, it alone cannot fully link these metabolites to their pharmacological effects *in vivo*. Similarly, systems biology approaches such as network pharmacology, while providing frameworks to explore disease-treatment mechanisms of traditional Chinese medicines, often lack direct connection to the actual bioactive components that enter the body (Li and Zhang, 2013). Serum pharmacochemistry further emphasizes that only metabolites entering the systemic circulation can serve as authentic pharmacodynamic substances (Feng et al., 2022; Ma et al., 2017), yet integrating this principle with both chemical identification and mechanistic exploration remains underexplored.

This study introduces a novel integrated strategy for the first time by combining high-resolution mass spectrometry (to pinpoint plasma-absorbed metabolites of Shenshuaifu granule as candidate pharmacodynamic substances) with *in silico* technology (to map their interactions with CRF-related targets and pathways). We bridge the gap between chemical component identification and mechanism explanation. This approach not only ensures the specificity of pharmacodynamic substances (based on serum exposure) but also enhances the accuracy of mechanistic prediction, addressing the limitations of single-method approaches and providing a more systematic understanding of Shenshuaifu granule’s therapeutic effects in CRF.

## 2 Materials and methods

### 2.1 Materials and reagents

Shenshuaifu granules (batch No.: 240317) were provided by the Fourth Clinical Medical College of Guangzhou University of Chinese Medicine. The chemical reference substances with purity ≥98% and HPLC grade, including stachydrine, betaine, caffeic acid, calycosin, chrysophanic acid, calycosin-7-O-beta-D-glucoside, cryptotanshinone, and alisol C 23-acetate, were purchased from China National Drug Control Institute (Beijing, China). Formic acid and acetonitrile (HPLC-grade) used for chromatographic analysis were purchased from Thermo Fisher Scientific Company (Massachusetts, United States). Deionized water was commercially obtained from Hangzhou Wahaha Group Limited (Hangzhou, China). All the other reagents, such as methanol, were of analytical grade and provided by Kermel Chemical Co., Ltd. (Tianjin, China).

### 2.2 Animals and treatment

Six male Sprague–Dawley specific pathogen-free (SPF) rats, aged 6–8 weeks, weighing 180–220 g, were purchased from Beijing Vital River Corporation (Beijing, China). The experimental procedure was carried out in strict accordance with the ethical regulations of Guangzhou University of Chinese Medicine (No. 2022038). The feeding environment conditions were set as follows: the temperature was 20–26°C, the humidity was 30%–70%, the light cycle was a 12-h/12-h cycle, and the animals could freely access food and water. Experiments were performed after the mice had acclimatized to the laboratory conditions for 1 week.

### 2.3 Plant metabolites of Shenshuaifu granule

#### 2.3.1 Preparation of the Shenshuaifu granule test solution

A weighted 1.0 g granule sample was ultrasonically extracted for 20 min with 20 mL of 70% methanol. The solution was filtered through 0.22-μm membrane filters. Then, the filtrate was diluted ten times with methanol as a test solution before analysis.

#### 2.3.2 Plasma sample preparation of Shenshuaifu granule

Six male SD rats were randomly divided into a blank group and a Shenshuaifu granule group, with three rats in each group. The rats fasted for 12 h before the experiment, except for water. Rats in the Shenshuaifu granule group were given Shenshuaifu granule dissolved in deionized water at a dose of 1.0 g/kg, while rats in the blank group were given the same volume of normal saline. Two hours after administration, the rats were intraperitoneally injected with 2% pentobarbital sodium 40 mg/kg to be deeply anesthetized. The whole blood was collected from the abdominal aorta and centrifuged. Then, the supernatant was taken and stored at −80°C.

A 200-μL sample of plasma was added to 800 μL of methanol in a 1.5-mL EP tube and vortex mixed for 3 min. After centrifugation at 12,000 rpm for 15 min at 4°C, the supernatant was transferred to a new 1.5-mL EP tube and dried under nitrogen. Then, the plasma residue samples were redissolved in 100 μL methanol. A 2-μL aliquot of the supernatant was injected into the UHPLC-QE-MS for analysis.

### 2.4 Apparatus and chromatographic conditions

The plant metabolite analysis of Shenshuaifu granule was performed on an UltiMate™ 3000 RSLCnano ultra-high-performance liquid chromatography system in tandem with Q-Exactive-Orbitrap mass spectrometry (UHPLC-QE-MS, Thermo Fisher Scientific, United States). The chromatographic separation was performed on a Waters Acquity UHPLC CSH C_18_ column (2.1 × 100 mm, 1.7 mm, Waters, United States) at a flow rate of 0.3 mL/min at 35°C. The mobile phase contained 0.1% formic acid aqueous solution (A) and acetonitrile (B). The following gradient elution procedure was used for chromatographic separation of Shenshuaifu granule test solution: 0–2 min, 5%–20% B; 2∼4min, 20%–50% B; 4–8 min, 50%–90% B; 8–10 min, 90%–99%; 10–13 min, 99%–99% B; 13–13.1 min, 99%–5%; 13.1–15 min, 5%B. The injection volume was 2 μL. The QE mass spectrometer was used to collect primary and secondary mass spectrometry data. The specific parameters were as follows: sheath gas was 40 ARB, auxiliary gas was 10 ARB, ion transfer tube temperature was 350°C, full MS resolution was 70,000, MS/MS resolution was 17,500, collision energy (in NCE mode) was 20/30/40, spray voltage was 3.5 kV (positive ion mode) or −3.0 kV (negative ion mode). The following gradient elution procedure was employed for the chromatographic separation of plasma samples of Shenshuaifu granule: 0–1.5 min, 5%–15%; 1.5–7 min, 15%–25%; 7–9 min, 25%–40%; 9–16 min, 40%–70%; 16–20 min, 70%–99%; 20–21.5 min, 99%–99%; 21.5–22 min, 99%–5%; 22–25 min 5%. The remaining chromatographic and mass spectrometry parameters were consistent with those utilized for the analysis of Shenshuaifu granule.

The database of plant metabolites from Shenshuaifu granule, including chemical names, chemical formulas, precursor ions, and secondary mass spectrometry information, was established by retrieving data from PubMed (https://pubmed.ncbi.nlm.nih.gov/), Web of Science (https://www.webofscience.com/wos/), and CNKI (https://c61.oversea.cnki.net/). Xcalibur 4.1.3.1 software (Thermo Fisher Scientific, United States) and Progenesis QI v3.0 software (Waters, United States) were used to analyze the first and second-order mass spectra of plant metabolites from Shenshuaifu granule.

### 2.5 Computational studies

#### 2.5.1 Screening of intersection targets

Intersection targets were screened between the nine prototype plant metabolites of Shenshuaifu granule detected in the rat plasma and renal failure. The information on plant metabolites in plasma was input into the SwissTargetPrediction database (http://www.swisstargetprediction.ch/). The related targets of plant metabolites were collected via the UniProt database (https://www.uniprot.org). Then, these targets were converted into a gene symbol to obtain the disease targets of CRF from the Gene Cards database (https://www.genecards.org/) with “synchronous real failure” as the keyword.

#### 2.5.2 Construction of the protein–protein interaction (PPI) network

The Venn map was drawn online by Venny 2.1.0 (https://bioinfogp.cnb.csic.es/tools/venny/index.html) to obtain the common potential targets of the plant metabolites of Shenshuaifu granule in plasma and CRF. The PPI network was built through the STRING Database (https://www.stringdb.org/). The above results were imported into the Cytoscape 3.10.1 software for visualization of the core targets of Shenshuaifu granule against CRF with degree, betweenness, and closeness as indicators.

#### 2.5.3 GO and KEGG enrichment analysis

The core targets of Shenshuaifu granule for CRF were entered into the DAVID database (https://david.ncifcrf.gov/) for GO and KEGG enrichment analyses. The top 10 biological processes, cellular components, molecular functions, and the top 20 key signaling pathways were screened at *p* < 0.05, respectively. The GO and KEGG enrichment analyses were visualized through the Wei Sheng Xin platform (https://www.bioinformatics.com.cn/).

#### 2.5.4 Construction of the “metabolite–target–pathway–disease” network

The “metabolite–target–pathway–disease” network was constructed by Cytoscape 3.10.1 software with the plant metabolites in plasma, potential targets, and the top 20 key signaling pathways obtained from KEGG enrichment analysis.

#### 2.5.5 Molecular docking analysis

Based on the results described in Section 2.5.4, five core plant metabolites were selected for molecular docking. The five core metabolites of Shenshuaifu granule in plasma were considered ligands, and the top 10 key targets were considered receptors. The RCSB PDB database (https://www.rcsb.org/#Category-welcome) was searched for the molecular structure files of receptors. The PubChem database (https://pubchem.ncbi.nlm.nih.gov/) was used to download the 2D structure of ligands, and the molecular docking was realized by AutoDock Vina (version: 1.1.2) (https://vina.scripps.edu/).

## 3 Results

### 3.1 Chemical analysis of Shenshuaifu granule

**FIGURE 1:**
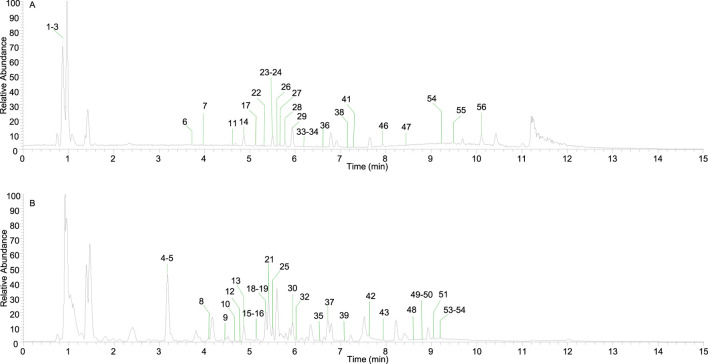
The total ion chromatogram of Shenshuaifu granule. **(A)** The total ion chromatogram in the positive ion mode of Shenshuaifu granule; **(B)** The total ion chromatogram in the negative ion mode of Shenshuaifu granule. The plant metabolites marked No. 1–56 in the chromatograms are shown in Table 1.

Figure 1 shows the total ion flow diagram of Shenshuaifu granule in positive and negative ion modes. In the mode of simultaneous scanning of positive and negative ions, a total of 56 plant metabolites were identified in the sample solution of Shenshuaifu granule, including 25 in the positive ion detection mode and 31 in the negative ion detection mode (Table 1). Among them, there are 11 phenolic acids, including ferulic acid and caffeic acid; 15 flavonoids, including hesperidin, phytomelin, and apigenin; 11 anthraquinones, including emodin, rhein, and emodin methyl ether; 10 saponins, including astragaloside III, alisol C, and soyasaponin I; and nine other classes, including betaine, tumulosic acid, and leonurine.

**TABLE 1:** Characterization of the plant metabolites of Shenshuaifu granule by UHPLC-QE-MS.

NO.	RT (min)	Formula	PI pred. (Da)	PI meas. (Da)	Error (ppm)	Ion mode	Fragment (m/z)	Identification
1	0.92	C_5_H_11_NO_2_	118.0863	118.0863	0.1	M + H	59.0730,72.0808,102.0550,116.0707,118.0863	Betaine
2	0.93	C_7_H_7_NO_2_	138.055	138.0546	−2.4	M + H	94.0651,120.0444	Trigonelline
3	0.97	C_7_H_13_NO_2_	144.1019	144.1017	−1.8	M + H	58.0652,144.1017	Stachydrine
4	3.17	C_9_H_8_O_4_	179.035	179.0341	−4.7	M-H	59.0138,71.0138,179.0350	Caffeic acid
5	3.17	C_9_H_10_O_5_	197.0455	197.0449	−3.4	M-H	109.0295,123.0451,135.0451,179.0350,197.0455	Salvianic acid A
6	3.66	C_28_H_32_O_16_	625.1763	625.176	−0.5	M + H	200.0680,241.0496,463.1235	Isorhamnetin-3-O-neohespeidoside
7	3.93	C_21_H_20_O_10_	433.1129	433.1126	−0.7	M + H	97.0285,283.00601,313.0707,337.0707,367.0813,397.0918	Emodin-8-glucoside
8	4.12	C_16_H_18_O_9_	353.0878	353.0885	2	M-H	71.0138,135.0451,191.0561,233.0455,353.0878	Chlorogenic acid
9	4.38	C_22_H_22_O_10_	445.114	445.1148	1.8	M-H	282.0533,310.0483,355.0823,430.0905	Calycosin-7-O-beta-D-glucoside
10	4.68	C_28_H_34_O_15_	609.1825	609.1841	2.7	M-H	135.0451,563.1770	Hesperidin
11	4.69	C_14_H_21_N_3_O_5_	312.1554	312.1557	0.9	M + H	72.0808,114.1026,132.1132,211.0601,236.0918,295.1289	Leonurine
12	4.77	C_27_H_30_O_16_	609.1461	609.1478	2.8	M-H	151.0037,609.1461	Phytomelin
13	4.84	C_15_H_10_O_5_	269.0455	269.0458	1	M-H	211.0400,240.0428	Apigenin
14	4.87	C_16_H_12_O_5_	285.0757	285.0757	−0.2	M + H	163.0386	Calycosin
15	5.16	C_21_H_20_O_11_	447.0933	447.0944	2.5	M-H	151.0037,255.0299,447.0933	Astragalin
16	5.18	C_36_H_30_O_16_	717.1461	717.148	2.6	M-H	109.0295,339.0510,519.0932	Salvianolic acid L
17	5.27	C_10_H_10_O_4_	195.0652	195.0652	0.1	M + H	117.0335,193.0496	Ferulic acid
18	5.3	C_18_H_12_O_7_	339.051	339.0512	0.5	M-H	280.0377,321.0404	Salvianolic acid G
19	5.31	C_27_H_22_O_12_	537.1038	537.1046	1.4	M-H	109.0295,313.0717,493.1140	Lithospermic acid
20	5.35	C_18_H_16_O_8_	359.0772	359.0785	3.6	M-H	123.0451,197.0455,359.0772	Rosmarinic acid
21	5.41	C_36_H_30_O_16_	717.1461	717.1469	1.1	M-H	109.0295,161.0244,339.0510,519.0932	Salvianolic acid B
22	5.43	C_21_H_18_O_11_	447.0922	447.0918	−0.8	M + H	73.0284,85.0284,97.0285,113.0234	Rhein-8-glucoside
23	5.51	C_16_H_12_O_4_	269.0808	269.0808	−0.2	M + H	223.0754,233.0598,269.0809	Formononetin
24	5.51	C_22_H_22_O_9_	431.1337	431.1335	−0.3	M + H	177.0546,431.1337	Ononin
25	5.57	C_17_H_14_O_6_	313.0718	313.072	0.7	M-H	59.0138,109.0281,135.0451,203.0350,239.0713,254.0584,313.0717	Salvianolic acid F
26	5.6	C_26_H_22_O_10_	495.1286	495.128	−1.1	M + H	181.0496,279.0652,314.0785	Salvianolic acid A
27	5.66	C_17_H_16_O_5_	301.1071	301.1072	0.4	M + H	191.0703,207.0652,301.1071	Methylnissolin
28	5.71	C_17_H_18_O_5_	303.1227	303.1223	−1.3	M + H	133.0648,193.0860,301.1071	Isomucronulatol
29	5.88	C_16_H_14_O_6_	303.0863	303.0859	−1.4	M + H	109.0284,135.0441,153.0546,163.0390,257.0809,303.0863	Hesperetin
30	5.91	C_15_H_10_O_7_	301.0354	301.0354	0.2	M-H	121.0295,151.0037,178.9986,273.0404	Quercetin
31	5.95	C_15_H_10_O_6_	285.0405	285.0404	−0.3	M-H	135.0087,269.0455	Kaempferol
32	6.02	C_15_H_10_O_5_	269.0455	269.0455	−0.1	M-H	109.0295,135.0451,225.0557,268.0377,269.0455	Emodin
33	6.24	C_41_H_68_O_14_	807.4501	807.4483	−2.3	M + Na	627.3867	Astragaloside IV
34	6.26	C_41_H_68_O_14_	785.4682	785.4664	−2.3	M + H	143.1067,455.3520	Astragaloside III
35	6.53	C_48_H_78_O_18_	941.5115	941.5121	0.6	M-H	205.0709	Soyasaponin I
36	6.63	C_43_H_70_O_15_	827.4787	827.4765	−2.7	M + H	125.0961,143.1067,157.0496,175.0601	Astragaloside II
37	6.72	C_15_H_10_O_4_	253.0506	253.0503	−1.4	M-H	224.0479,225.0557,252.0429,253.0506	Chrysophanic acid
38	7.14	C_32_H_48_O_6_	529.3524	529.3514	−1.8	M + H	227.1431,353.2475,469.3313,529.3524	Alisol C 23-acetate
39	7.15	C_15_H_10_O_5_	269.0455	269.0455	−0.2	M-H	147.0451,240.0428	Baicalein
40	7.22	C_15_H_8_O_6_	283.0258	283.0258	3.5	M-H	239.0349,268.0377,283.0248	Rhein
41	7.42	C_45_H_72_O_16_	869.4893	869.4873	−2.3	M + H	97.0285,125.0961,217.0707	Astragaloside I
42	7.64	C_30_H46O5	485.3272	485.327	−0.6	M-H	387.2541,427.2854,485.3272	Alisol C
43	7.88	C_30_H_46_O_5_	485.3272	485.3271	−0.2	M-H	367.2642,423.3268	Poricoic acid G
44	7.95	C_15_H_10_O_5_	269.0455	269.0453	−0.9	M-H	240.0428	Aloe-emodin
45	7.99	C_16_H_12_O_5_	283.0612	283.061	−0.6	M-H	268.0378,283.0612	Physcion
46	8.01	C_30_H_50_O_5_	513.355	513.3561	2.2	M + Na	399.2894,513.355,229.1653	Alisol A
47	8.45	C_18_H_14_O_3_	279.1016	279.1012	−1.5	M + H	169.0648,261.0911	Dihydrotanshinone I
48	8.56	C_31_H_46_O_6_	513.3222	513.3219	−0.5	M-H	325.2173,449.3061	Peroxydehydrotumulosic acid
49	8.75	C_30_H_44_O_5_	483.3116	483.3117	0.2	M-H	409.2748	Poricoic acid B
50	8.76	C_18_H_12_O_3_	275.0714	275.0712	−0.6	M-H	275.0713	Tanshinone I
51	9.08	C_31_H_46_O_5_	497.3272	497.3269	−0.7	M-H	423.2904	Poricoic acid A
52	9.13	C_30_H_48_O_4_	471.348	471.3476	−0.8	M-H	453.3374	Alisol B
53	9.18	C_31_H_50_O_4_	485.3636	485.3632	−0.9	M-H	423.3268	Tumulosic acid
54	9.25	C_19_H_20_O_3_	297.1485	297.1479	−2.1	M + H	237.0911,279.1380	Cryptotanshinone
55	9.49	C_32_H_50_O_5_	515.3731	515.3718	−2.5	M + H	151.1118,219.1744,437.3415,515.3731	Alisol B 23-acetate
56	10.07	C_19_H_18_O_3_	295.1329	295.1322	−2.4	M + H	226.0938,277.1224	Tanshinone IIA

### 3.2 Analysis of plant metabolites of Shenshuaifu granule in rat plasma

**FIGURE 2:**
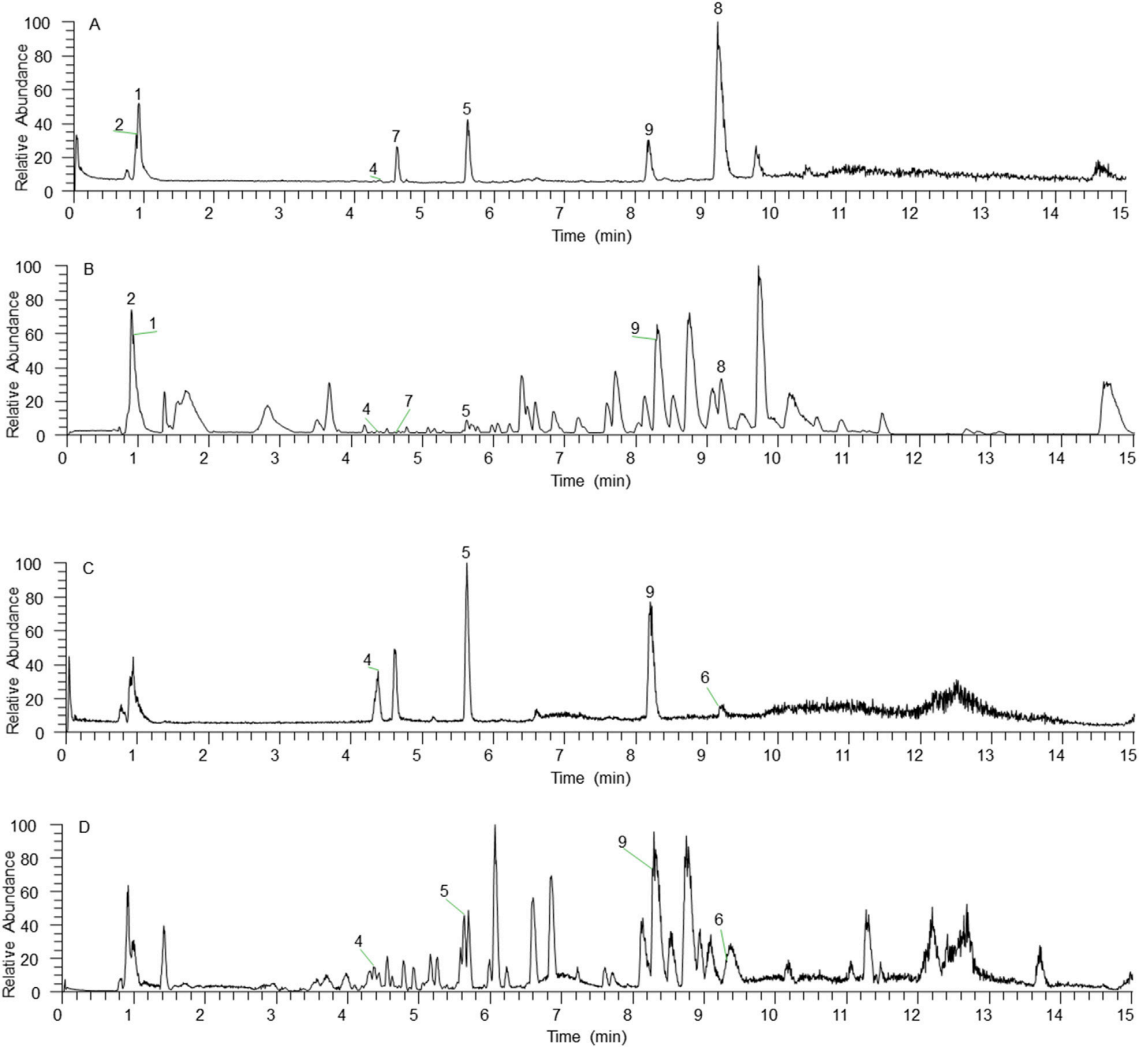
The total ion chromatogram of plant metabolites of Shenshuaifu granule absorbed into the rat plasma. **(A)** The total ion chromatogram in the positive ion mode of the mixed reference solution; **(B)** The total ion chromatogram in the positive ion mode of the plant metabolites of Shenshuaifu granule absorbed into the rat plasma; **(C)** The total ion chromatogram in the negative ion mode of the mixed reference solution; **(D)** The total ion chromatogram in the negative ion mode of the plant metabolites of Shenshuaifu granule absorbed into the rat plasma. The plant metabolites corresponding to numbers 1–9 are shown in Table 2.

In the mode of simultaneous scanning of positive and negative ions, nine plant metabolites from Shenshuaifu granule were determined from the rat drug-containing plasma (Table 2; Figure 2). Eight prototype plant metabolites of Shenshuaifu granule in rat plasma were identified by comparison with the chemical reference solution. Among them, there are two quinones, two flavonoids, two phenols, two alkaloids, and one terpene.

**TABLE 2:** The prototype plant metabolites of Shenshuaifu granule absorbed into the blood circulation.

No.	RT (min)	Formula	Ion mode	Mass (m/z)	Identity	Source
1	0.84	C_7_H_13_NO_2_	M + H	144.1018	Stachydrine	*Leonurus japonicus* herb
2	0.85	C_5_H_11_NO_2_	M + H	118.0862	Betaine	*Lycium chinense* fruit
3	5.87	C_21_H_18_O_11_	M + H	447.0924	Rhein-8-glucoside	*Rheum palmatum* root and rhizome
4	6.25	C_9_H_8_O_4_	M-H	179.0351	Caffeic acid	*Salvia miltiorrhiza* root and rhizome
5	6.75	C_16_H_12_O_5_	M + H	285.0759	Calycosin	*Astragalus membranaceus* root
6	8.81	C_15_H_10_O_4_	M-H	253.0506	Chrysophanic acid	*Rheum palmatum* root and rhizome
7	10.34	C_22_H_22_O_10_	M-H	445.1142	Calycosin-7-O-beta-D-glucoside	*Astragalus membranaceus* root
8	17.21	C_19_H_20_O_3_	M + H	297.1468	Cryptotanshinone	*Salvia miltiorrhiza* root and rhizome
9	18.85	C_32_H_48_O_6_	M + Na	551.3309	Alisol C 23-acetate	*Alisma orientale* rhizome

### 3.3 Identification of the plant metabolites active against CRF

#### 3.3.1 Potential targets and PPI network construction

A total of 274 targets related to the nine plant metabolites of Shenshuaifu granule in plasma were found in the Swiss target prediction database. There were 5430 CRF-related targets in the Gene Cards database. There are 209 common potential targets between the plant metabolites of Shenshuaifu granule in plasma and CRF (Supplementary Figure S1). In PPI networks, the larger the size of the node, the darker the color of the node, and the more important the gene is. A total of 41 targets were obtained in Supplementary Figure S2, among which TNF, SRC, STAT3, EGFR, BCL2, JUN, PTGS2, ESR1, HSP90AA1, and MPP9 may be the key targets of Shenshuaifu granule against CRF.

#### 3.3.2 GO and KEGG enrichment analysis

**FIGURE 3:**
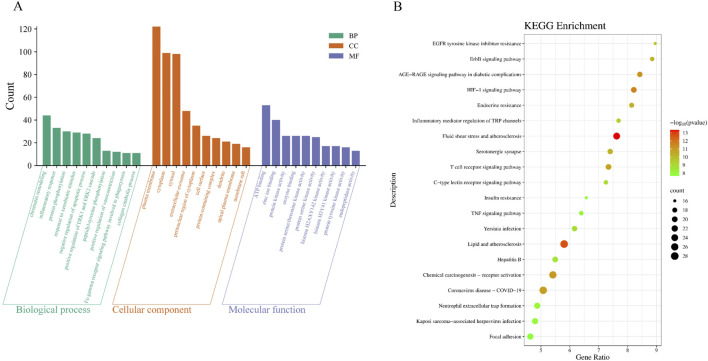
The potential signaling pathways of Shenshuaifu granule against chronic renal failure. **(A)** GO enrichment analysis of Shenshuaifu granule against chronic renal failure. **(B)** KEGG enrichment analysis of Shenshuaifu granule against chronic renal failure.

According to the result of Section 3.3.1, GO and KEGG enrichment analyses were performed on 209 potential targets of CRF treated by the granule. The enrichment results of GO were sorted from small to large according to the *p*-value, and Figure 3A shows the data visualization of taking the top 10 relevant entries of biological process, cellular component, and molecular function. KEGG enrichment analysis yielded 167 entries. The top 20 signaling pathways were mainly enriched in the lipid and atherosclerosis pathways, the AGE-RAGE signaling pathway in diabetic complications, the HIF-1 signaling pathway, the TNF signaling pathway, etc. (Figure 3B; Supplementary Table S1).

#### 3.3.3 “Metabolite–target–pathway–disease” network construction

**FIGURE 4:**
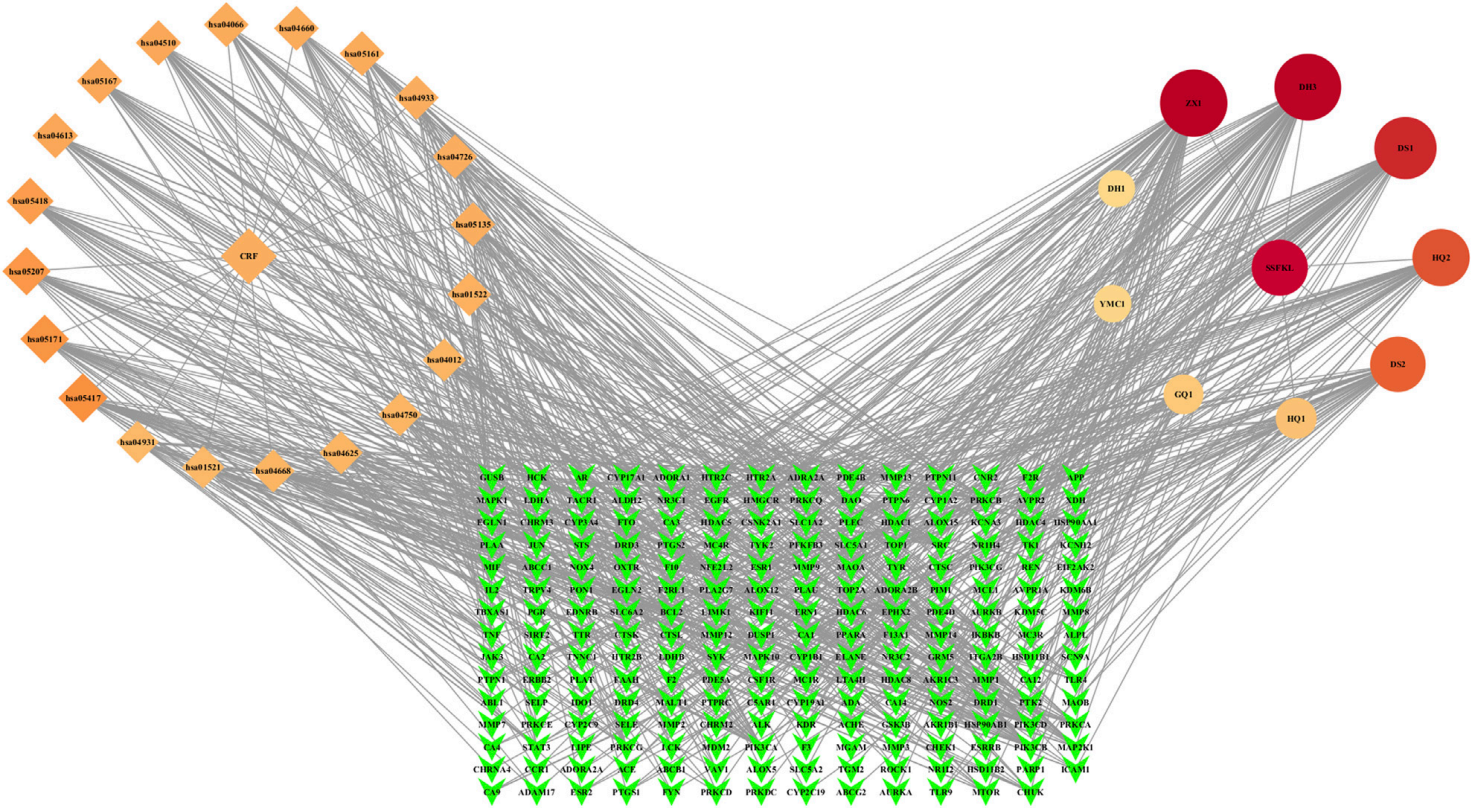
“Metabolite–target–pathway–disease” network of Shenshuaifu granule against chronic renal failure. The diamonds identify the top 20 pathways from KEGG enrichment analysis; the circular shapes identify the nine plant metabolites absorbed into the blood of rats; the green quadrilateral shapes identify 209 potential targets. CRF: chronic renal failure; SSFKL: Shenshuaifu granule; DH1: rhein-8-glucoside; DH3: chrysophanic acid; DS1: cryptotanshinone; DS2: caffeic acid; GQ1: betaine; HQ1: calycosin-7-O-beta-D-glucoside; HQ2: calycosin; YMC1: stachydrine; ZX1: alisol C 23-acetate.

The nine plant metabolites of Shenshuaifu granule in plasma, 209 intersection targets, and the top 20 signaling pathways obtained from KEGG enrichment analysis were imported into the software to construct a network diagram (Figure 4). The results predicted that alisol C 23-acetate, chrysophanic acid, cryptotanshinone, calycosin, and caffeic acid were the five core metabolites of Shenshuaifu granule in blood against CRF.

#### 3.3.4 Molecular docking verification

**FIGURE 5:**
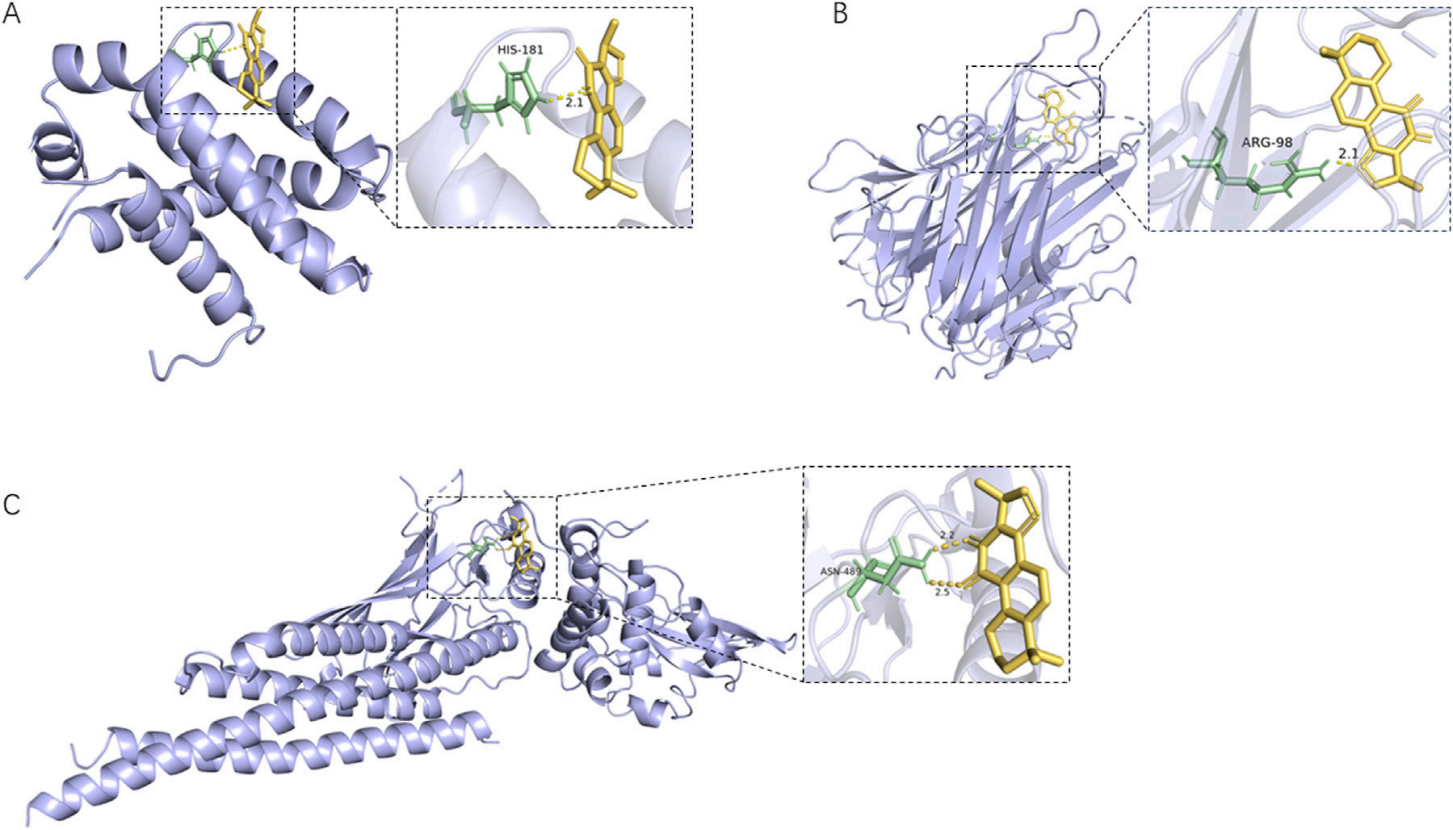
The active cavity of different target molecules docking with cryptotanshinone. **(A)** Docking result of cryptotanshinone as a BCL2 inhibitor; **(B)** Docking result of cryptotanshinone as a TNF inhibitor; **(C)** Docking result of cryptotanshinone as a STAT3 inhibitor.

The five core plant metabolites in the blood above and the top 10 key targets, including TNF, SRC, STAT3, EGFR, BCL2, JUN, PTGS2, ESR1, HSP90AA1, and MMP9, were subjected to molecular docking. The binding activities were determined according to the binding energy. The lower the binding energy, the stronger the binding ability. As shown in Supplementary Figure S3, the binding energy of the core plant metabolites of Shenshuaifu granule in plasma with core targets is ≤ −4.5 kcal/mol. Among them, the binding energy of most plant metabolites was even lower than −7 kcal/mol, indicating that these metabolites have a tight binding relationship with the 10 key targets. For example, an active cavity of the target allowed cryptotanshinone, as a small-molecule inhibitor, to bind well to the targets, as shown in Figure 5.

## 4 Discussion

CRF is a worldwide public health problem with increased prevalence (Olsen and van Galen, 2022). In recent years, Chinese medicines have made great progress in the treatment of CRF (Liao et al., 2024; Zheng et al., 2024; Zou et al., 2024). The curative effect of Shenshuaifu granule on CRF has been proven in the clinic. In this study, UHPLC-QE-MS was used to identify the plant metabolites in Shenshuaifu granule as well as the plant metabolites in the plasma after administration of Shenshuaifu granule. Then, *in silico* studies were performed to predict the core targets and signaling pathways and to preliminarily verify the mechanisms at the end.

The plant metabolites from Shenshuaifu granule have been reported to have kidney protective effects. This finding provides a scientific basis for exploring the active compounds of botanical drugs and supporting clinical applications. For example, betaine has effects in the treatment of kidney disease, including reducing homocysteine levels, anti-inflammatory and antioxidant properties, and protecting kidney function. Betaine supplementation can reduce the levels of creatinine and urea nitrogen in plasma and reduce kidney injury (Alvarenga et al., 2022; McRae, 2013). In diabetes-induced renal injury, caffeic acid protects renal function by reducing serum creatinine and blood urea nitrogen and inhibiting the production of inflammatory cytokines (such as IL-1β, IL-18, IL-6, and TNF-α) and the expression of NLRP3 inflammasome (Akhlaghipour et al., 2023). Some clinical trials have shown that rhein can reduce the serum levels of triglyceride, cholesterol, and TGF-β1 in patients with diabetic nephropathy, reduce proteinuria, blood urea nitrogen, and serum creatinine, and improve renal function (Zeng et al., 2014). Cryptotanshinone has a protective effect on renal ischemia–reperfusion injury by inhibiting inflammatory response and renal cell apoptosis and regulating the NF-κB and p38 MAPK signaling pathways to improve renal function and structure (Bai et al., 2019).

Pharmaceutical compounds usually work through the blood circulation. In this study, the plant metabolites of Shenshuaifu granule in plasma were selected as the research object to conduct a network pharmacology study, to establish the “metabolite–target–pathway–disease” network, and to provide a more reliable basis for pharmacodynamic mechanism exploration. We predicted that flavonoids and quinones in Shenshuaifu granule are potential active plant metabolites in the granule that could exert anti-CRF through specific targets and pathways, which are consistent with the functions of reported targets and pathways. For example, both EGFR and SRC family kinases are involved in the TGF-β signaling pathway, while TGF-β is a key process in the development of renal fibrosis (Arbesú et al., 2017; Dongre and Weinberg, 2019). PTGS2 is also known as COX-2. In kidney diseases, the overexpression of COX-2 is related to the development of glomerular sclerosis and fibrosis, and COX-2 inhibitors are used to improve renal function (Ferrer et al., 2019). In addition, TNF is closely related to glomerular damage and sclerosis. TNF causes renal fibrosis by excessive deposition of extracellular matrix (Varfolomeev et al., 2012). STAT3 is considered to be a promising therapeutic target for a variety of kidney diseases, including diabetic nephropathy, acute kidney injury, lupus nephritis, polycystic kidney disease, and renal cell carcinoma (Bienaimé et al., 2016; Yu et al., 2023). KEGG enrichment analysis showed that Shenshuaifu granule could treat CRF through an AGE-RAGE signaling pathway. The binding of AGEs to their receptor RAGE activates oxidative stress and inflammatory responses, such as the activation of the NF-κB signaling pathway, which in turn promotes the progression of kidney damage (Wu et al., 2021). Directly blocking the AGE-RAGE signaling pathway and reducing inflammatory responses leads to protecting kidney function (Zhu et al., 2024).

Finally, the good interaction between the plant metabolites of Shenshuaifu granule in plasma and the targets screened by molecular docking preliminarily verified the accuracy of the active plant metabolites and targets found in this study, as well as the mechanism of prediction. The above findings provide a reliable reference and direction for future studies on the mechanism of Shenshuaifu granule *in vitro* and *in vivo* in the treatment of CRF. In the next step, we will further validate the active compounds and the pharmacological mechanism of Shenshuaifu granule discovered in this study through *in vitro* and *in vivo* experiments. It will also be possible to verify the quality assurance of this comprehensive strategy.

## 5 Conclusion

In this study, 56 chemical compounds, especially nine prototype plant metabolites in rat plasma, were successfully identified from Shenshuaifu granule for the first time. This crucial discovery not only enriches the understanding of the chemical composition of Shenshuaifu granule but also lays a solid foundation for in-depth research on its active material basis, as these plasma-existing prototype metabolites are likely to be the key substances exerting pharmacological effects *in vivo*. Further results indicated that flavonoids and quinones in Shenshuaifu granule were the potential active compounds, suggesting anti-inflammatory, anti-glomerulosclerosis, and fibrosis effects on CRF. Finally, this study performed a completely new comprehensive strategy (high-resolution mass spectrometry combined with *in silico* technology) to establish the connection between chemical components and mechanisms, providing new ideas for the study of the active ingredients and mechanisms of traditional Chinese medicines.

## Data Availability

The original contributions presented in the study are included in the article/Supplementary Material; further inquiries can be directed to the corresponding author.
