# Supplementary figures and images for: Unveiling the material basis of Shenshuaifu granule and its therapeutic mechanism in chronic renal failure: a combined approach of high-resolution mass spectrometry and in silico technology
Source: Front Chem. 2025 Aug 20;13:1563598. doi: 10.3389/fchem.2025.1563598 (PMC12405226; doi:10.3389/fchem.2025.1563598)

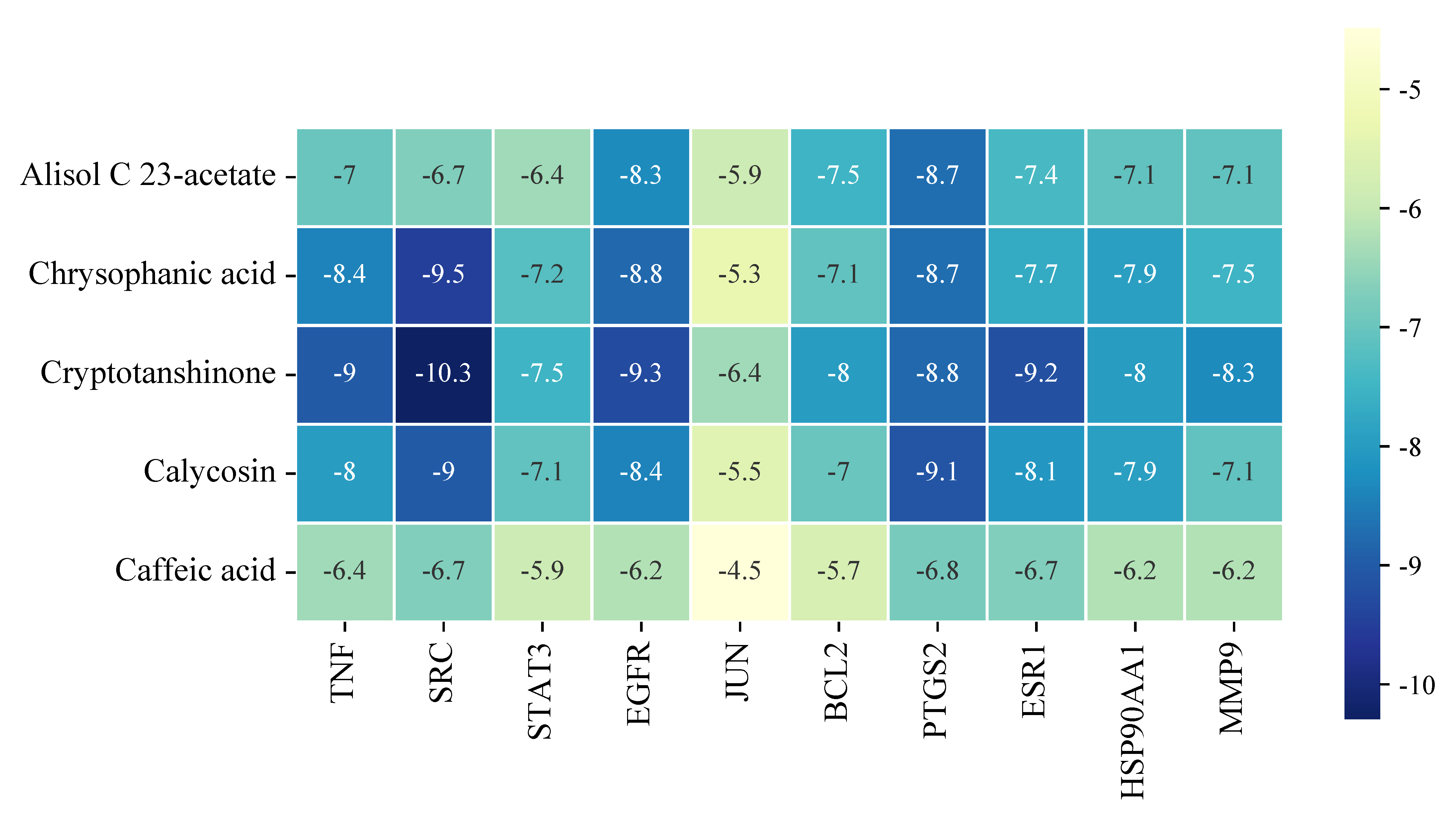

Supplement: Supplementary file 1 [file Image3.tiff]

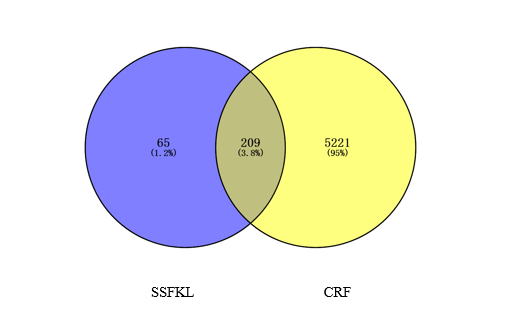

Supplement: Supplementary file 2 [file Image1.tiff]

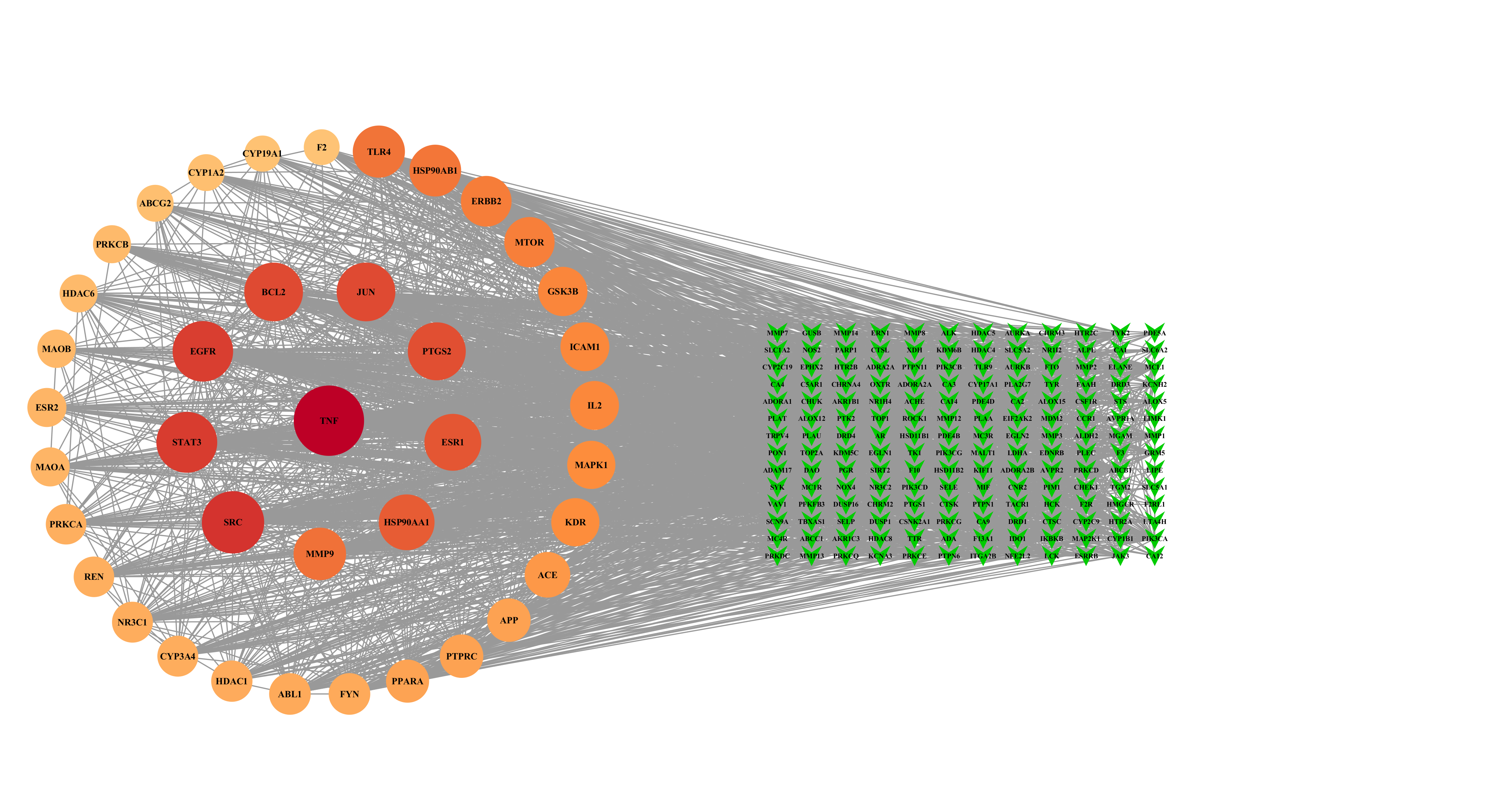

Supplement: Supplementary file 4 [file Image2.tiff]
