# Supplementary material for: Unveiling the material basis of Shenshuaifu granule and its therapeutic mechanism in chronic renal failure: a combined approach of high-resolution mass spectrometry and in silico technology
Source: Front Chem. 2025 Aug 20;13:1563598. doi: 10.3389/fchem.2025.1563598 (PMC12405226; doi:10.3389/fchem.2025.1563598)
Supplement: Supplementary file 3 [file Supplementaryfile1.docx]

**Supplementary materials:**

**Unveiling the Material Basis of Shenshuaifu G****ranule and Its Therapeutic Mechanism in Chronic Renal Failure: A Combined Approach of High-Resolution Mass Spectrometry and** **In Silico Technology**

**Huang Qingbao^1^, Xian Leyao^2^, Song Xiansheng^2^, Zou Dawei^2^, Chen Junqi^1^, Chen Yue^1^, Li Wanquan^1^, Zhang Shangbin^1^, Liang Huichao^1,*^**

1. Shenzhen Key Laboratory of Hospital Chinese Medicine Preparation, Shenzhen Traditional Chinese Medicine Hospital, The Fourth Clinical Medical College of Guangzhou University of Chinese Medicine, Shenzhen, 518033, PR China
2. Chinese Medicine Guangdong Laboratory, The Second Affiliated Hospital of Guangzhou University of Chinese Medicine, Guangzhou, 510120, P.R. China

*Corresponding author:

E-mail addresses: 515191980@qq.com (Liang Huichao)

**Results of supplementary materials**

**Figures：**

Figure S1. Venn diagram of the prototype plant metabolites of Shenshuaifu granule in rat plasma and disease targets of chronic renal failure. SSFKL: Shenshuaifu granule; CRF: chronic renal failure.

Figure S2. The potential targets of Shenshuaifu granule against chronic renal failure by PPI analysis.

Figure S3 The molecular docking results of core plant metabolites and core targets on Shenshuaifu granule against chronic renal failure.

**Tables：**

Table S1. KEGG enrichment analysis of Shenshuaifu granule for the treatment of chronic renal failure

| **ID** | **Description** | **P Value** | **Count** | **Gene** |
| --- | --- | --- | --- | --- |
| hsa05418 | Fluid shear stress and atherosclerosis | 4.20635E-14 | 24 | JUN, HSP90AA1, HSP90AB1, CHUK, DUSP1, SRC, MMP2, ITGA2B, PIK3CD, PLAT, PIK3CB, SELE, TNF, MMP9, PTK2, ICAM1, MAPK10, IKBKB, PIK3CA, CTSL, TRPV4, KDR, BCL2, NFE2L2 |
| hsa05417 | Lipid and atherosclerosis | 1.89002E-13 | 28 | GSK3B, HSP90AB1, SRC, PIK3CD, PIK3CB, TNF, ICAM1, IKBKB, MAPK1, JUN, HSP90AA1, CHUK, MMP1, MMP3, STAT3, PRKCA, SELE, MMP9, VAV1, PTK2, SELP, ERN1, MAPK10, CYP2C9, PIK3CA, BCL2, TLR4, NFE2L2 |
| hsa04066 | HIF-1 signaling pathway | 2.26035E-12 | 20 | PRKCG, EGLN1, MAP2K1, EGLN2, PFKFB3, NOS2, PRKCB, STAT3, PIK3CD, PRKCA, PIK3CB, EGFR, MTOR, LDHB, LDHA, PIK3CA, ERBB2, BCL2, MAPK1, TLR4 |
| hsa04933 | AGE-RAGE signaling pathway in diabetic complications | 5.95273E-12 | 19 | JUN, PRKCB, PRKCE, MMP2, PRKCD, STAT3, PIK3CD, PRKCA, PIK3CB, SELE, F3, TNF, ICAM1, MAPK10, PIK3CA, PIM1, BCL2, NOX4, MAPK1 |
| hsa05207 | Chemical carcinogenesis - receptor activation | 8.19115E-12 | 26 | HSP90AB1, CHRNA4, SRC, PIK3CD, PIK3CB, CYP3A4, EGFR, CYP1B1, MAPK1, PRKCG, JUN, MAP2K1, HSP90AA1, PRKCB, EPHX2, STAT3, PRKCA, ESR1, MTOR, ESR2, AR, PIK3CA, CYP1A2, BCL2, PGR, PPARA |
| hsa05171 | Coronavirus disease - COVID-19 | 1.28251E-11 | 27 | C5AR1, PIK3CD, F13A1, PIK3CB, TNF, EGFR, IKBKB, MAPK1, PRKCG, JUN, ACE, SYK, CHUK, PRKCB, MMP1, MMP3, STAT3, EIF2AK2, PRKCA, TYK2, F2, IL2, SELP, MAPK10, ADAM17, PIK3CA, TLR4 |
| hsa04660 | T cell receptor signaling pathway | 1.80226E-11 | 20 | GSK3B, MAP2K1, JUN, CHUK, PIK3CD, PTPN11, PIK3CB, TNF, IL2, MALT1, VAV1, MAPK10, IKBKB, PTPRC, PIK3CA, LCK, MAPK1, FYN, PTPN6, PRKCQ |
| hsa01522 | Endocrine resistance | 4.39856E-11 | 18 | MAP2K1, JUN, SRC, MMP2, PIK3CD, PIK3CB, ESR1, MMP9, EGFR, MTOR, PTK2, ESR2, MAPK10, PIK3CA, ERBB2, MDM2, BCL2, MAPK1 |
| hsa04012 | ErbB signaling pathway | 4.74592E-11 | 17 | PRKCG, GSK3B, MAP2K1, JUN, PRKCB, SRC, PIK3CD, PRKCA, PIK3CB, EGFR, MTOR, PTK2, MAPK10, PIK3CA, ERBB2, ABL1, MAPK1 |
| hsa04726 | Serotonergic synapse | 5.7718E-11 | 19 | PRKCG, APP, MAP2K1, MAOB, MAOA, PRKCB, DUSP1, ALOX15, HTR2B, HTR2C, PRKCA, ALOX12, HTR2A, PTGS2, CYP2C19, PTGS1, CYP2C9, ALOX5, MAPK1 |
| hsa01521 | EGFR tyrosine kinase inhibitor resistance | 1.74371E-10 | 16 | PRKCG, GSK3B, MAP2K1, PRKCB, SRC, STAT3, PIK3CD, PRKCA, PIK3CB, EGFR, MTOR, PIK3CA, ERBB2, KDR, BCL2, MAPK1 |
| hsa04750 | Inflammatory mediator regulation of TRP channels | 4.30827E-10 | 17 | PRKCG, PRKCB, SRC, PRKCE, PRKCD, HTR2B, PIK3CD, HTR2C, PRKCA, ALOX12, PIK3CB, HTR2A, MAPK10, PIK3CA, TRPV4, F2RL1, PRKCQ |
| hsa04625 | C-type lectin receptor signaling pathway | 1.06146E-09 | 17 | JUN, SYK, CHUK, SRC, PRKCD, PIK3CD, PTPN11, PIK3CB, PTGS2, TNF, IL2, MALT1, MAPK10, IKBKB, PIK3CA, MDM2, MAPK1 |
| hsa05135 | Yersinia infection | 1.25482E-09 | 19 | GSK3B, MAP2K1, JUN, ROCK1, CHUK, SRC, LIMK1, PIK3CD, PIK3CB, TNF, IL2, VAV1, PTK2, MAPK10, IKBKB, PIK3CA, LCK, MAPK1, TLR4 |
| hsa05161 | Hepatitis B | 2.94304E-09 | 20 | PRKCG, MAP2K1, JUN, CHUK, PRKCB, SRC, STAT3, PIK3CD, PRKCA, TYK2, PIK3CB, TNF, MMP9, MAPK10, IKBKB, PIK3CA, BCL2, MAPK1, JAK3, TLR4 |
| hsa04668 | TNF signaling pathway | 6.94266E-09 | 17 | MAP2K1, JUN, CHUK, MMP3, PIK3CD, PIK3CB, PTGS2, SELE, TNF, MMP9, ICAM1, MAPK10, IKBKB, MMP14, ADAM17, PIK3CA, MAPK1 |
| hsa04613 | Neutrophil extracellular trap formation | 8.70941E-09 | 21 | PRKCG, HDAC4, HDAC5, MAP2K1, SYK, PRKCB, HDAC1, SRC, C5AR1, ITGA2B, PIK3CD, PRKCA, PIK3CB, HDAC8, MTOR, HDAC6, SELP, PIK3CA, MAPK1, TLR4, ELANE |
| hsa05167 | Kaposi sarcoma-associated herpesvirus infection | 1.13597E-08 | 21 | CCR1, GSK3B, MAP2K1, JUN, SYK, CHUK, SRC, STAT3, EIF2AK2, PIK3CD, TYK2, PIK3CB, PTGS2, MTOR, PIK3CG, ICAM1, MAPK10, IKBKB, HCK, PIK3CA, MAPK1 |
| hsa04931 | Insulin resistance | 1.51572E-08 | 16 | PTPN1, GSK3B, PRKCB, PRKCE, PRKCD, STAT3, PIK3CD, PTPN11, PIK3CB, TNF, MTOR, MAPK10, IKBKB, PIK3CA, PRKCQ, PPARA |
| hsa04510 | Focal adhesion | 2.07874E-08 | 21 | PRKCG, GSK3B, MAP2K1, JUN, ROCK1, PRKCB, SRC, ITGA2B, PIK3CD, PRKCA, PIK3CB, EGFR, VAV1, PTK2, MAPK10, PIK3CA, ERBB2, KDR, BCL2, MAPK1, FYN |
